# Recommendations for measuring and standardizing light for laboratory mammals to improve welfare and reproducibility in animal research

**DOI:** 10.1371/journal.pbio.3002535

**Published:** 2024-03-12

**Authors:** Robert J. Lucas, Annette E. Allen, George C. Brainard, Timothy M. Brown, Robert T. Dauchy, Altug Didikoglu, Michael Tri H. Do, Brianna N. Gaskill, Samer Hattar, Penny Hawkins, Roelof A. Hut, Richard J. McDowell, Randy J. Nelson, Jan-Bas Prins, Tiffany M. Schmidt, Joseph S. Takahashi, Vandana Verma, Vootele Voikar, Sara Wells, Stuart N. Peirson

**Affiliations:** 1 Centre for Biological Timing, School of Biological Sciences, Faculty of Biology Medicine and Health, University of Manchester, Manchester, United Kingdom; 2 Department of Neurology, Thomas Jefferson University, Philadelphia, Pennsylvania, United States of America; 3 Department of Structural and Cellular Biology, Tulane University School of Medicine, Tulane, Louisiana, United States of America; 4 Department of Neuroscience, Izmir Institute of Technology, Gülbahçe, Urla, Izmir, Turkey; 5 F.M. Kirby Neurobiology Center and Department of Neurology, Boston Children’s Hospital and Harvard Medical School, Center for Life Science, Boston, Massachusetts, United States of America; 6 Novartis Institute for Biomedical Research, Cambridge, Massachusetts, United States of America; 7 Section on Light and Circadian Rhythms (SLCR), National Institute of Mental Health, John Edward Porter Neuroscience Research Center, Bethesda, Maryland, United States of America; 8 RSPCA, Horsham, West Sussex, United Kingdom; 9 Chronobiology Unit, Groningen Institute of Evolutionary Life Sciences, University of Groningen, Groningen, the Netherlands; 10 Department of Neuroscience, Rockefeller Neuroscience Institute, West Virginia University, Morgantown, West Virginia, United States of America; 11 The Francis Crick Institute, London, United Kingdom; 12 Leiden University Medical Centre, Leiden, the Netherlands; 13 Department of Neurobiology, Northwestern University, Evanston, Illinois, United States of America; 14 Department of Neuroscience, Peter O’Donnell Jr Brain Institute, University of Texas Southwestern Medical Center, Dallas, Texas, United States of America; 15 Howard Hughes Medical Institute, University of Texas Southwestern Medical Center, Dallas, Texas, United States of America; 16 NASA Ames Research Center, Space Biosciences Division, Moffett Field, California, United States of America; 17 Laboratory Animal Center and Neuroscience Center, HiLIFE, University of Helsinki, Helsinki, Finland; 18 The Mary Lyon Centre, MRC Harwell, Harwell Campus, Oxfordshire, United Kingdom; 19 Sleep and Circadian Neuroscience Institute (SCNi), Kavli Institute for Nanoscience Discovery, Nuffield Department of Clinical Neurosciences, University of Oxford, Oxford, United Kingdom

## Abstract

Light enables vision and exerts widespread effects on physiology and behavior, including regulating circadian rhythms, sleep, hormone synthesis, affective state, and cognitive processes. Appropriate lighting in animal facilities may support welfare and ensure that animals enter experiments in an appropriate physiological and behavioral state. Furthermore, proper consideration of light during experimentation is important both when it is explicitly employed as an independent variable and as a general feature of the environment. This Consensus View discusses metrics to use for the quantification of light appropriate for nonhuman mammals and their application to improve animal welfare and the quality of animal research. It provides methods for measuring these metrics, practical guidance for their implementation in husbandry and experimentation, and quantitative guidance on appropriate light exposure for laboratory mammals. The guidance provided has the potential to improve data quality and contribute to reduction and refinement, helping to ensure more ethical animal use.

## Abstract 2

Lighting conditions for laboratory mammals is currently set according to the sensitivity of human vision. This Consensus View defines alternative ‘animal-centric’ metrics and provides guidance for their application to standardize experimental conditions, improve animal welfare and the quality of animal research.

## Introduction

**Fig 1:**
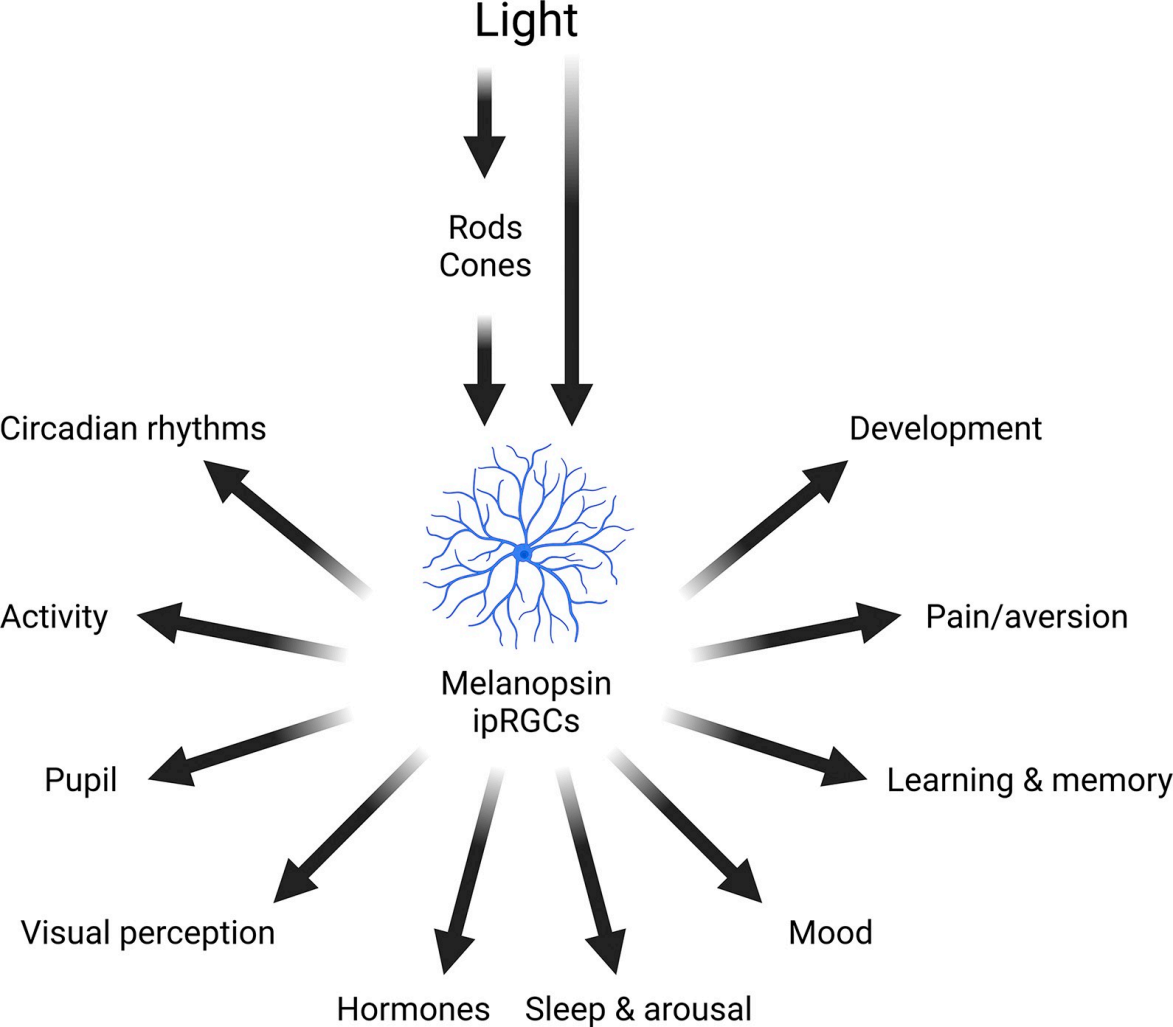
Effects of light on mammalian physiology and behavior. As well as mediating vision, light detected by rods, cones, and melanopsin ipRGCs modulate a wide range of different physiological and behavioral responses. Figure redrawn from [6]. Created with BioRender.com. ipRGC, intrinsically photosensitive retinal ganglion cell.

Light has wide-ranging effects on mammalian biology (Fig 1). In addition to supporting vision [1], light impacts numerous body systems and behavioral and physiological processes, either directly or via its effects on the circadian clock [2]. All life is exposed to a rhythmically changing cycle of day and night, produced by the rotation of the Earth on its axis. As a result, the light intensity from the sun can vary by around 10 orders of magnitude over the course of the day [3]. As well as changes in light intensity, at dawn and dusk the spectrum of the light environment also changes with a progressive enrichment of shorter wavelengths due to atmospheric scatter and filtering [4,5]. All animals possess an endogenous circadian clock, enabling them to anticipate predictable changes in their environment. A clock, however, must be set to the correct time: a process termed entrainment. In mammals, light provides the primary time cue for entrainment [6–8], as well as regulating accessory visual responses such as pupil constriction [9]. Environmental light also modulates light adaptation via retinal circuits to alter visual perception [10,11]. As circadian rhythms regulate processes throughout the body, light exposure has the potential to modulate numerous aspects of physiology and behavior beyond those that directly respond to light [12].

Light also regulates hormone release, including pineal melatonin [13] and adrenal glucocorticoids [14]. Sleep and arousal are modulated by light, with nocturnal light exposure in rodents leading to rapid sleep induction [15–18]. A large body of work has shown that light regulates mood and a range of cognitive processes [19–23]. More recently, studies have shown that light can also regulate nociception [24,25]. And finally, the light environment may exert an important role during development, including neonatal aversive behaviors [26,27], retinal vasculature development [28], ocular growth to ensure emmetropia [29,30], and synapse formation in the brain [31]. Together, these data illustrate the wide-ranging influence of light on mammalian physiology and behavior. These responses are mediated predominantly by a combination of retinal rods, cones, and melanopsin-expressing intrinsically photosensitive retinal ganglion cells (ipRGCs) (Fig 1).

It follows that lighting is an important consideration in laboratory animal husbandry and experimentation, which should be measured and regulated appropriately, yet ambient light for animals is typically quantified in units (lux) designed for human observers (Box 1). In this Consensus View, we discuss and present the most appropriate metrics for the quantification of light appropriate for nonhuman mammals in husbandry and experimental settings. We also provide guidance on how to measure and implement these metrics in a practical setting.

**Box 1. The measurement problem**

Light is defined as that portion of the electromagnetic spectrum visible to the human eye. The fundamentally species-specific nature of this definition should lead us to question its suitability for other animals and, indeed, it is widely appreciated that some species can use radiation outside the human sensitivity range (ultraviolet) for vision. Perhaps, less widely known is that the human-oriented definition of light is also fundamental to the way in which it is quantified. The System International (SI) is the official system of measurement that forms the basis of scientific, technical, and industrial measurements worldwide and consists of 7 base units of measurement. The SI base unit for light, the candela, quantifies light according to its apparent brightness for a standard human observer. As other commonly used lighting metrics, including lumens and lux (the unit for ambient light intensity), are derivatives of the candela, it follows that almost all light quantification currently assumes a human observer.

## Methodology

Building on the success of previous meetings addressing measurement and recommendations for human light exposure [2,32], Robert Lucas and Stuart Peirson convened a third International Workshop on Circadian and Neurophysiological Photometry held in Manchester, United Kingdom in 2023 to address the problem of light measurement in laboratory animal research. Workshop participants (authors of this Consensus View) were identified on the basis of professional and/or academic qualifications (accounting for COVID-19-related travel restrictions), encompassing expertise in retina-driven effects of light in laboratory mammals, animal husbandry, and welfare. The stated goals of the workshop were to: agree on measures to replace illuminance (photopic lux) and human color descriptors in quantifying the laboratory mammal light experience; consider the tools required to make those quantities widely measurable; and provide quantitative recommendations for healthy light exposure for laboratory mammals during the day and at night. We retained a focus on measures of ambient light (rather than local intensity or visual contrast) as the most relevant parameter for animal housing and for influential circadian, neuroendocrine, and neurobehavioral effects of light. We limited our objectives to mammals because non-mammalian vertebrates have a much wider array of photoreceptor types (including extra-retinal photosensitivity), making the task of species-specific light measurement substantially more complex.

Participants were sent a briefing document and a recorded presentation prepared by Lucas and Peirson in advance, which defined the problem of light measurement for animals and described how the recently standardized metrology of α-opic irradiance could be adapted to use across species [33]. The meeting itself began with topic-relevant presentations from participants and discussion of the α-opic metrology; there was unanimous agreement that α-opic metrology was the best available approach for species-specific measurement. Participants then split into 4 working groups addressing: standardizing measures across species; describing “color”; target values for husbandry; and practical challenges to implementation. Working group discussions were followed by a period of feedback to the whole community for general discussion and consensus. At each stage, time was allowed for all opinions to be voiced and for review of the relevant literature where appropriate. Working groups then devised a plan to draft elements of this Consensus View, which were submitted to the chairs (Lucas and Peirson) for integration into a complete draft that was reviewed, edited, and approved by all workshop participants.

## Species-specific quantification of ambient light intensity

### Background

**Fig 2:**
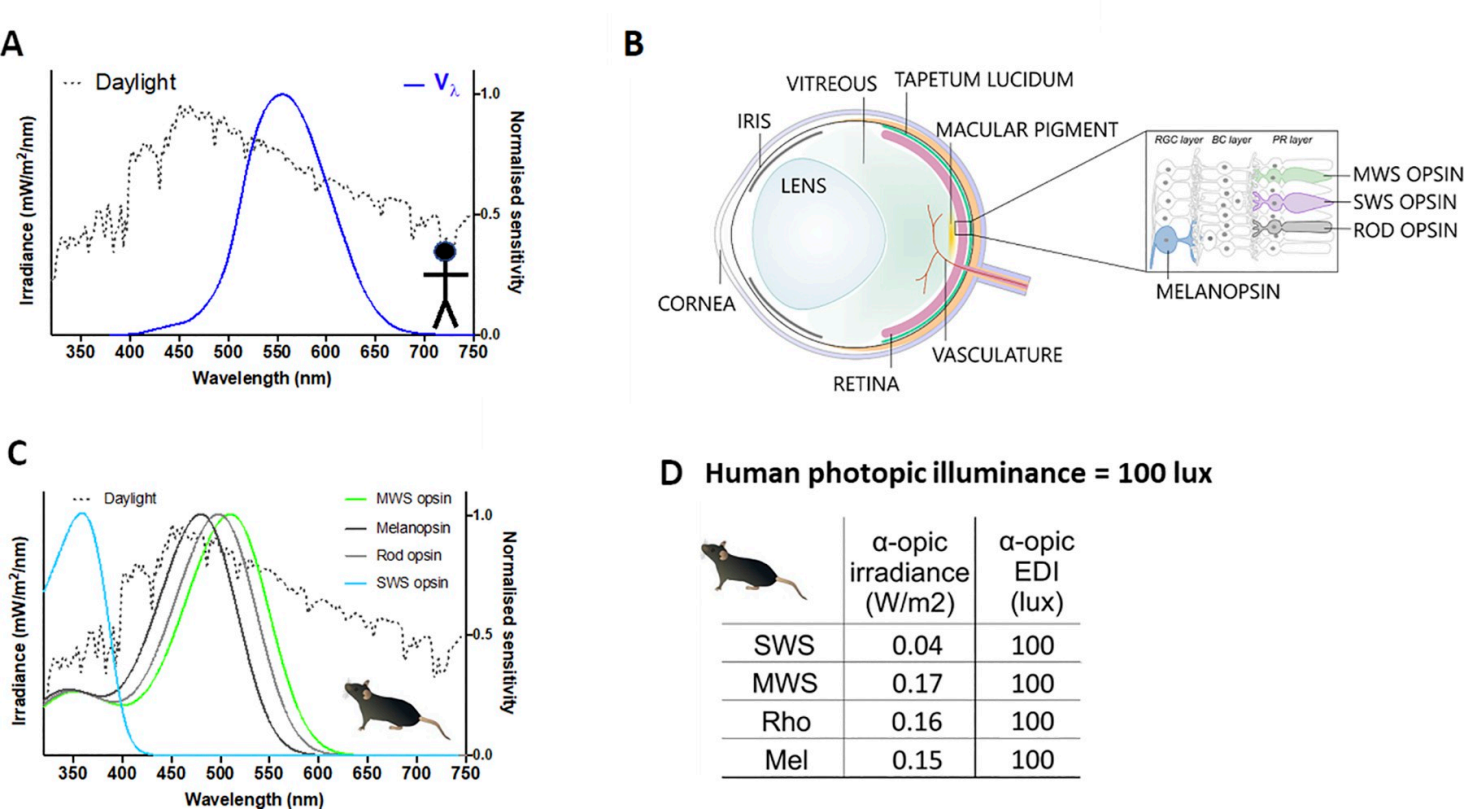
Quantifying light using spectral weighting functions. (**A**) Illuminance in photopic lux is calculated by weighting power across the spectrum according to a function that describes the wavelength sensitivity of perceived brightness in humans (Vλ, blue line). (**B**) A species-specific approach to quantification aims to calculate effective intensity not for a particular visual endpoint, such as perceived brightness, but for each of the 4 types of mammalian retinal photopigment (melanopsin, rod opsin, SWS, and MWS cones). The in vivo spectral sensitivity of these photopigments is defined by their intrinsic wavelength preference and the “pre-receptoral” filter applied by elements of the eye that impact light reaching them (labeled on the schematic of a prototypical mammalian eye). Note species may differ in the complement of photopigments and pre-receptoral filters. (**C**) In vivo spectral weighting functions (S1 Text) for each photopigment in mouse shown as a representative (note divergence from Vλ; **A**). (**D**) Ambient light intensity for mice may be quantified in 4 α-opic irradiances or α-opic EDIs by applying photoreceptor spectral weighting functions (**C**) to spectral power density measures. Here, values for the representative daylight spectrum (dotted line in **A** and **B**) are set at 100 photopic lux. EDI, equivalent daylight illuminance; MWS, middle-wavelength sensitive; SWS, short-wavelength sensitive.

The anthropomorphic nature of the candela (and derivatives including lux) arises from the fact that light can vary not only in total energy but also in how that energy is distributed across wavelengths. As humans are not equally sensitive to all wavelengths, simply summing energy across the spectrum cannot predict its apparent brightness. Rather, a spectral efficiency function (known as the photopic sensitivity function or V(λ)), defined according to the wavelength sensitivity of an assay of human perceived brightness, must first be applied (Fig 2A). V(λ) peaks at 555 nm, far from the portion of the spectrum to which many animals are most sensitive. Consequently, lights differing in spectral power distribution could have different effective brightness for laboratory animals, even if matched for a human observer. For these reasons, the current use of anthropomorphic metrics is suitable neither for describing light as experienced by these mammals in experimentation or husbandry, nor for agreeing on quantitative guidelines for light exposure.

In principle, species-specific versions of photopic lux could be created by replacing V(λ) with an equivalent description of spectral sensitivity for perceived brightness for the organism in question. We consider this neither practicable nor necessarily desirable. V(λ) is defined according to psychophysical brightness matching paradigms that would be arduous to reproduce across species. Moreover, it aims to predict only one aspect of visual perception (achromatic brightness) and is not appropriate, even in humans, for circadian, neuroendocrine, and neurobehavioral effects that involve melanopsin and have a more wide-ranging impact on behavior and physiology [2,34]. For these reasons, we considered species-specific versions of a more recently standardized human metrology based on the concept of α-opic irradiance [33].

The α-opic irradiance metrology (Box 2) was developed to update metrics to account for circadian and related neurophysiological light responses, whose spectral sensitivity is not well approximated by V(λ) even in humans. Wavelength weighting functions in this approach are defined by the spectral sensitivity not of any single visual response (as is the case for V(λ)), but rather of the light sensitive proteins (photopigments) responsible for detecting light. At present, a reasonable simplification holds that mammalian neurophysiological light responses begin with photon absorption by the rod opsin, cone opsin, and melanopsin photopigments found in rod, cone, and ipRGC photoreceptors (Fig 2B and 2C). We therefore concentrate here on the problem of quantifying light as experienced by these photoreceptors. The fundamental approach we describe is scalable to additional photopigments as necessary. In particular, the mammalian genome contains 2 additional opsins, *Opn3* and *Opn5* [35,36], that have been linked to physiological responses [37–43]. OPN5, at least, is capable of acting as a photopigment. At present, their contribution to mammalian photobiology is less thoroughly elucidated than for the other photopigments, and both are expressed also outside of the retina where light filtering by overlying tissue would change wavelength sensitivity (S1 Text). As mice lacking rods, cones and melanopsin lack major light responses we therefore argue that concentrating on these best characterized photoreceptors is a reasonable first step to standardizing measurement.

**Box 2. α-Opic irradiance/equivalent daylight illuminance**

α-Opic irradiance quantifies light according to its effective intensity for each retinal photopigment separately. The method for calculating α-opic irradiance for each of the human photopigments (rhodopsin; short-, medium-, and long-wavelength sensitive cone opsins; and melanopsin) has recently been standardized for humans [33]. Each of these photopigments absorbs light according to its own spectral sensitivity profile, and hence, each will provide its own distinct response to light intensity for a given spectrum. That means that by integrating the photopigment’s spectral sensitivity profile with the spectral power distribution of incident light, it is possible to calculate an α-opic irradiance (Eq 1) that describes “effective” irradiance experienced for that photoreceptor system (Fig 2). Importantly, the α-opic irradiance concept is readily translatable across species [2], as it can be calculated for any photopigment in any species for which spectral sensitivity information is available.

$$E_{\alpha \beta }=\int E_{e,\lambda }(\lambda )s_{\alpha \beta }(\lambda )d\lambda$$ Eq 1

Where: *E*_*αβ*_ is the α-opic irradiance (that is, the irradiance for a given photopigment (α) in a given species (β), with units in W/m^2^); *E*_*e*,*λ*_(*λ*) is the spectral power distribution measured at the cornea, with units in W/m^2^; and *s*_*αβ*_(*λ*) is the spectral sensitivity of a given photopigment in a given species, corrected for pre-receptoral filtering.

α-Opic irradiance quantifies light in effective energy per unit area (W/m^2^). Further processing allows expression in the more intuitive quantity of α-opic equivalent daylight illuminance (EDI). α-Opic EDI describes the quantity of daylight (in photopic lux) required to produce that α-opic irradiance [33]. To convert α-opic irradiance to α-opic EDI, the α-opic efficacy of luminous radiation (ELR) is first defined by dividing the α-opic irradiance of a standard sunlight spectrum (termed D65) by its illuminance (in photopic lux). α-Opic EDI is then produced by multiplying α-opic irradiance by α-opic ELR (Eq 2).

$$E_{\alpha \beta }^{D65}=\frac{E_{\alpha \beta }}{K_{\alpha \beta }^{D65}}$$ Eq 2

Where: $E_{\alpha \beta }^{D65}$ is the α-opic EDI for a photopigment (α) in a species (β), with units lux β α-opic EDI, and $K_{\alpha \beta }^{D65}$ is the α-opic efficacy of luminous radiation for a photopigment in a species.

The advantage of expressing light in terms of α-opic EDI is that measures across photoreceptors, and indeed species, can be described in terms of a common, ethologically relevant anchor (an amount of daylight). The danger of α-opic EDI is that its unit (lux) is the same as for the currently used human-oriented photopic measurement system (even though it is calculated in a quite different way). To minimize the potential for confusion, we propose that the units for α-opic EDI are modified to incorporate the species and photoreceptor (e.g., lx mouse mel EDI or lx rat short-wavelength sensitive (SWS) EDI).

Note that the α-opic metrology quantifies light according to its effective intensity. It makes no assumptions about the photoreceptor response to that stimulus, which would depend on activation threshold, saturation point, and irradiance response function.

### Working group recommendation

Our working group agreed that α-opic irradiance was the most appropriate starting point for quantifying ambient light across mammalian species. For most mammals, it would reduce the spectral power distribution to 4 α-opic quantities (for rhodopsin, S-cone opsin, M-cone opsin, and melanopsin; Fig 2D), which represent the building blocks for all physiological light responses. As these quantify effective intensity for each photopigment, they represent the minimum number of values required to fully describe the animal’s experience. In principle, the α-opic metrology can also be adapted to measure effective radiance [33], but our discussions concentrated on its use to describe ambient light intensity (irradiance).

The core of the α-opic irradiance metrology is the photoreceptor-specific spectral efficiency function, *s*_*αβ*_(*λ*) which replaces V(λ) as a method of weighting energy across wavelength. We have provided *s*_*αβ*_(*λ*) functions for common species used in research in S1 Table. Given the central importance of *s*_*αβ*_(*λ*) in this metrology, we also provide a detailed description of the considerations and assumptions adopted in defining these functions and some guidance on extending the α-opic irradiance concept to other species (S1 Text). Application of *s*_*αβ*_(*λ*) to spectral power distributions returns effective optical power. A further feature of the α-opic metrology is a method of expressing this in a more familiar quantity. α-Opic EDI describes the quantity of daylight (in photopic lux) required to produce that α-opic irradiance (Box 2). α-Opic EDI thus allows intuitive comparisons of effective light intensity with a natural stimulus (daylight) in familiar units.

In principle, the α-opic measurement system is appropriate for any photoreceptor in any organism for which *s*_*αβ*_(*λ*) can be defined. The advantages of accurate quantification of effective intensity for polychromatic light provided by this measurement system are thus available to any animal (and indeed any photosensitive organism). We restricted the bulk of our discussions to mammals for simplicity as photoreception is often more complex in non-mammals. Amphibia, birds, reptiles, and fish have many (often >10) photopigment classes [44,45], with many of these pigments expressed outside the eye. As light reaching extra-retinal photoreceptors is filtered as it passes through overlying tissues, *s*_*αβ*_(*λ*) for a given extra-retinal photoreceptor may vary according to its location in the body. These complexities mean that a large number of α-opic quantities would be required to capture the full animal experience. Nonetheless, the α-opic system represents an excellent solution for individual or small groups of photoreceptors with defined *s*_*αβ*_(*λ*), which we hope relevant research communities will exploit.

## Guidance for measuring α-opic quantities in practice

**Fig 3:**
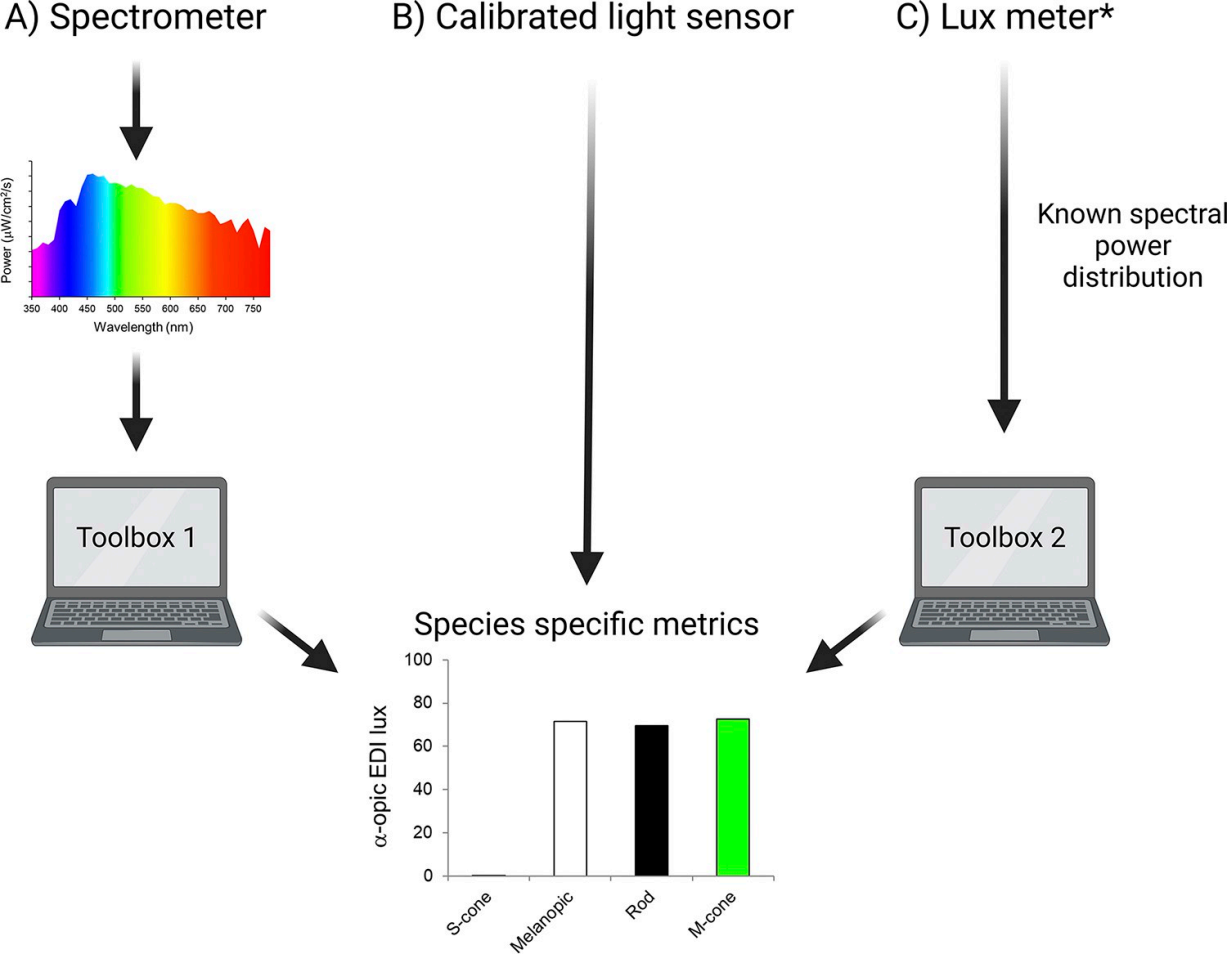
Methods for measuring species-specific α-opic EDIs. (**A**) Lx α-opic EDI can be determined from spectral power when measured by an appropriate spectrometer. We provide Toolbox 1 to calculate species-specific metrics from these measurements. (**B**) The most straightforward approach to measuring species-specific α-opic EDIs would be to use a light meter capable of returning light in these values. (**C**) A simple approach would be to estimate α-opic EDIs from measured photopic illuminance (output of lux meter) and knowledge of light source type, for which we provide Toolbox 2. *NB: this method will be less accurate and still requires the spectral power distribution of the light source to be known. Created with BioRender.com. EDI, equivalent daylight illuminance.

Although the mathematical procedure for calculating α-opic irradiance is straightforward, simple-to-use light meters working in these units are not widely available at the time of writing. We therefore next considered how these quantities could be measured in practice. The most conceptually straightforward, and accurate, approach is to use an optical spectrometer to measure the spectral power distribution of light (ideally measured at animal eye level) and apply mathematical conversions based upon Eq 1 (Box 2) to calculate α-opic irradiances (Fig 3). To facilitate such a process, we direct the reader to an online tool that will calculate species-specific α-opic irradiances/EDIs from input spectral power distributions based upon the *s*_*αβ*_ functions in S1 Table [46]. Sufficiently accurate spectrophotometers are available at moderate cost (>$500), but although relatively easy to use, may be intimidating for those unfamiliar with quantifying light. Moreover, this approach may become unwieldy when multiple measurements are required; for example, when describing light in various locations within a rack that has cages for animals at different levels.

The optimal tool for measuring α-opic irradiances would be a cheap, widely available, light meter that returns the relevant metrics without the user having to “peer beneath the bonnet” to see the underlying calculations. We encourage the lab supply industry to develop these. A simple design could integrate a spectrophotometer with suitable data processing capacity. Alternatively, cheap multichannel light sensors, which are increasingly being applied to measure human α-opic irradiances [47–49], could be recalibrated to measure species-specific metrics [46]. Examples of commercially available light meters and spectrophotometers are provided in S2 Table.

Mindful of the need to provide an accessible solution based upon currently available technology, a final possibility is to approximate α-opic irradiances using an estimate of spectral power distribution based upon the type of light source and its intensity. To this end, we present a rodent irradiance toolbox [2] that includes an option to convert photometer-based photopic lux measurements to species-specific units, provided that the spectrum of the illuminant (light source) is known (Fig 3). This is the least accurate of the options, as it assumes that the light reaching the detector has a spectral distribution matching the standard for that type of light source, unaltered by transmission or reflectance. Nevertheless, this option represents an opportunity to describe the animal experience more closely than is achievable using only photopic lux. Importantly, it can also be used to extract α-opic information from published studies, provided that they reported the light source used.

## Guidance for using α-opic measurements in animal husbandry and experiments

Having considered how light may be quantified, we turn our attention to direct advice on how this new metrology could be used to improve animal husbandry and experimentation [50]. Our advice is summarized in Box 3 and elucidated below.

**Box 3. Summary of guidance**

1. For conditions in which light is an aspect of experimental conditions:
  - Report α-opic equivalent daylight illuminance (EDI) in all relevant quantities.
  - Work towards agreeing on standardized conditions for your field of study.
2. For general animal husbandry:
  - Quantify and report light in melanopic EDI (units = lx melanopic EDI).
  - Provide a stable 24 h variation in light intensity, with light in the animals’ “night” being <0.1 lx melanopic EDI, and light during the “day” being >10 lx melanopic EDI. Light should be measured in the middle of the cage by pointing the detector in the direction of the major light source (usually upwards) and remembering that this guidance is for the animal and that colored caging may alter the spectrum of the animals’ light exposure. These targets should be reached in species-specific melanopic EDI, but human melanopic EDI is a reasonable approximation for indoor housing of at least some mammalian species under electric light without a strong output at <400 nm.
  - Animals should have the opportunity to escape light, for example, by retreating to a shelter and/or building an enclosed nest, which will require adequate nesting material.
3. For lighting designers, engineers, and architects:
  - Work towards cost and energy effective ways of achieving husbandry targets.
  - Consider lighting that provides lab animals with an approximation of their experience of daylight spectrum.
4. For laboratory equipment suppliers:
  - Provide light meters capable of measuring species-specific α-opic quantities.
  - Research and consider the impact of cage colors on animal’s light experience and work towards normalizing animal lighting experience across the rack.

### Experimentation

The most complete description of experimental conditions would encompass a complete quantification of light as experienced by the animal. This can be achieved by reporting species-specific α-opic irradiance (or EDI) for each photoreceptor. Ideally, this would be provided in methods sections both for general housing and, where appropriate, experimental conditions.

We were aware that quantifying α-opic irradiances lacks the simplicity of a single metric (c.f. photopic lux). For most lab mammals, 4 α-opic values would be required. This complexity reflects biology, as not only do light-evoked responses typically reflect a weighted output from all photoreceptive systems, but these weightings may differ across physiological outputs, or indeed between species. Applying the α-opic methodology to quantify light as experienced by individual photoreceptors removes those uncertainties and is the only way to capture the animal’s full experience. Moreover, our view is that this approach will itself provide a framework to better describe the photoreceptor origins of the myriad biological effects of light, in an approach that is transferable and comparable between species (and has already happened for humans) [2,32]. Finally, reporting all α-opic measures provides information about both effective irradiance and color.

The resources available (see above) mean that reporting light in 4 dimensions need not be onerous. Nevertheless, we also considered the additional problem of this quantification when it comes to recreating experimental conditions, as it is all but impossible to simultaneously match intensity across 4 α-opic dimensions. This complexity is unavoidable when applying light as an experimental parameter and should be accounted for in study design. For more general applications, however, it would be very helpful to have a single target metric when standardizing measurements or replicating experimental conditions. The answer to the question of which α-opic quantity to adopt for this purpose may differ according to the nature of the experiment, but as a rule of thumb we suggest using melanopic EDI. This choice is partly to retain consistency with the guidance for husbandry (see below). Furthermore, the similarity in spectral sensitivity between melanopsin and rods means that melanopic and rhodopic irradiance are strongly correlated across light sources. Consequently, under most circumstances melanopic irradiance will represent a good approximation of effective intensity for the retinal photoreceptors with lowest (rod) and highest (melanopsin) activation thresholds [51]. Matching melanopic irradiance may not always be sufficient to normalize experimental conditions (for example, when using lights of very divergent spectral power distribution), but melanopic irradiance will have much greater tolerance than the current practice of matching photopic lux.

### Animal husbandry

In the case of laboratory rodent husbandry, we feel that there is sufficient information to go beyond recommending that light is appropriately quantified and documented, and to provide some quantitative recommendations for light exposure. Many factors were considered in determining these, including circadian biology, light preference and aversion, human health and safety, and the animal’s species-specific experience. The guidance we provide is for light as experienced by the animal, and it is important to note that this will be determined not just by the nature of room lighting, but also by rack orientation, cage location within the rack, and cage color [52–54]. For this reason, the figures we give relate to in-cage light measurements, with the detector pointing towards the major light source (and the cage in its position in the rack if appropriate). The guidelines in Box 3 are based on available information, but this evidence base is certainly incomplete, and guidelines may evolve as new data are presented.

The first decision in defining healthy levels of lighting is which metric to provide targets for. As mentioned above, a complete description of the animal experience requires quantification in all α-opic irradiances. We note that there is good evidence that circadian and related neurophysiological responses can be engaged by all photoreceptors in laboratory rodents [9,55–61], and hope that the α-opic metrology will facilitate studies aimed at resolving their contribution to factors relevant for husbandry. Nevertheless, given the substantial practical advantages to using a single metric, we provide guidance here in terms of melanopic irradiance. Several factors persuaded us that this quantity could be applied to achieve a reasonable approximation of the animal experience. First, melanopsin-expressing ipRGCs are responsible for important determinants of animal welfare, including circadian photoentrainment and light-induced changes in physiological and behavioral states [6,8]. Secondly, as melanopsin cells have lower sensitivity than rods and comparable sensitivity to cones [51], as well as a spectral sensitivity in the short to middle wavelength portion of the visible range, any light sufficient to engage melanopsin will also be sufficient to support vision. Furthermore, as outlined above, the similarity in spectral sensitivity between melanopsin and rods means that melanopic irradiance would quantify light with sufficient accuracy across the full range of intensities to which mammals respond.

We propose a further simplification in order to facilitate adoption of guidelines. Although α-opic irradiances are species specific, we suggest using human melanopic irradiance as an acceptable shorthand for general animal husbandry. There is a danger of inaccuracy, but this is largely a problem when using very colored lights. Across a range of broad-spectrum lights (encompassing all commonly used room lighting), the median difference between human and mouse melanopic irradiance is only 7% (range 1% to 19%) (S3 and S4 Tables). Meanwhile, the increasing availability of light meters capable of measuring human melanopic irradiance makes it easy for any vivarium to compare their lighting against guidance specified in that measurement unit.

Turning to guidelines, we aimed for separate recommendations for the animal’s subjective daytime and night (Box 3). As complete darkness at night is neither natural nor easily achievable, we considered how much light animals might be exposed to at night in nature. The brightest natural light source at night is the moon. Although a bright super-moon can provide 0.3 photopic lux, Kyba and colleagues proposed 0.1 photopic lux as a more realistic value for moonlight [62]. We therefore suggest that light exposure during the dark phase should not exceed 0.1 lx human melanopic EDI (applying the approximation that photopic illuminance and melanopic EDI are near interchangeable for moonlight). Given the ethological basis for our decision, we believe this is a reasonable target for nighttime lighting for all mammalian species. To achieve these light levels, researchers and animal care staff will need to use dim red lighting for nighttime monitoring or welfare checks (see below).

Determining appropriate levels for daytime light is more complex. In principle, a similarly ethological approach could be taken by recommending that all animals have access to irradiances equivalent to natural daylight during the day. However, achieving such high irradiances is impractical in terms of human user experience and energy usage. We turned therefore to consider the minimum acceptable light exposure during the day. A useful starting point is the minimum intensity required for circadian entrainment. The circadian clock integrates light over long timeframes (tens of minutes [63]) and we considered a threshold here for a day (light) phase lasting at least several hours. For mice housed with dark nights, this can be very low, with entrainment reported for daytime light as low as 0.06 lx mouse melanopic EDI [64]. A more realistic target to ensure robust entrainment in all visually intact animals is 0.6 to 6 lx mouse melanopic EDI [55]. Moreover, several commonly used mouse strains have outer retinal degeneration, and the available evidence is that thresholds for entrainment are higher in animals with dysfunctional outer retina (at 6 lx mouse melanopic EDI [65]). We therefore suggest a minimum irradiance of 10 lx human melanopic EDI, which is roughly equivalent to the experience of civil twilight [5] and is much lower than the 250 lx human melanopic EDI recently recommended for humans [32].

We appreciate that 10 lx melanopic EDI is low compared to daylight and may be insufficient to fully engage the impact of light on physiological/behavioral states. The thresholds for circadian entrainment upon which it is based come from animals whose night phase is totally dark, and the impact of low light exposure in subjective night on thresholds for entrainment is not well established [66]. Moreover, the characteristics of circadian entrainment may also depend upon daytime light over a wider range [67–69]. Finally, as the threshold of 10 lx melanopic EDI is based on data from mice, it may be less appropriate for more distantly related and/or diurnal species (see e.g., data on diurnal rodents [67,70]). For these reasons, while we believe this value is supported by available evidence (and should be achievable without imposing large increases in energy usage), we stress that it should be viewed as a minimum that may be insufficient to fully normalize the animal experience and, where possible, brighter light is preferable.

We do not provide a recommended upper limit for daytime light intensity. Vivarium lighting will always be dimmer than daylight and thus fall within the range of natural light exposure. Nocturnal rodents typically avoid light when faced with a choice [71], and we recommend that cages contain a retreat space or shelter [72] and/or sufficient, suitable nesting material to allow them to do so [73]. Concerns are often raised about the potential for retinal damage under higher light intensities. For normal pigmented animals, the light intensities required to cause retinal damage are very high (>10,000 photopic lux) [74]. Where albino animals are used, current evidence suggests light levels should not exceed 20 photopic lux (corresponding to approximately 10 to 20 lx melanopic EDI, depending upon the light source) to avoid retinal damage [75].

## Further considerations

### Species-specific consideration of “color”

Thus far we have considered the challenge of quantifying and regulating ambient light intensity across mammalian species. But the experience of light is also determined by its spectral composition, a property humans perceive as color. In common with the general propensity to design, apply, and report lighting according to human perception, the spectral quality of animal lighting is typically designed with humans in mind. Specifically, by providing “white” light that gives objects a naturalistic color appearance, it aims to create the perceptual qualities of daylight for humans. By contrast, an animal’s experience of this lighting environment may deviate substantially from their experience of natural daylight.

**Fig 4:**
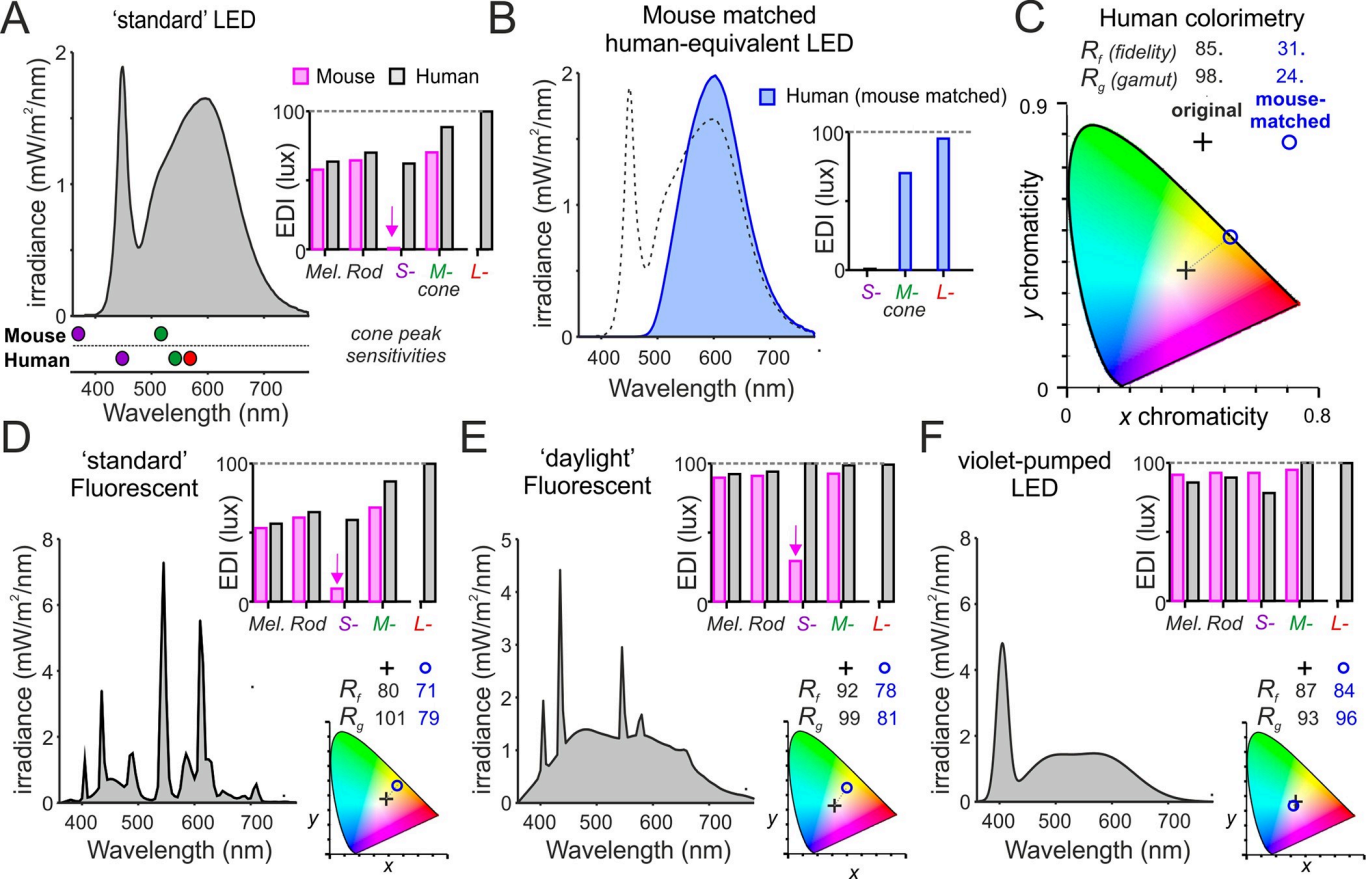
Predicted “color” qualities of different white light sources for mice. (**A**) Spectral power distribution of a standard 4000K white LED (100 photopic lux) and the corresponding α-opic EDIs for human and mouse. Lower panel shows peak sensitivities of mouse and human cone photoreceptors. Note the very low S-cone-opic EDI for mice due to the lack of energy at wavelengths <400 nm (>100-times less than for natural daylight of the same photopic illuminance). (**B**) To describe the impact of this difference for color experience in mice, we created a nominal spectral power distribution that recreates, in humans, the relative S- and M-cone-opic EDIs experienced by mice under light source in (**A**). (**C**) We then compared the x,y chromaticities (plotted on CIE 1931 2° color space) and IES-TM30 color-rendering metrics for color-fidelity (R_f_) and gamut (R_g_) for a standard human observer of the original white LED (from **A**) and the nominal light recreating the mouse experience (from **B**). Note the strong yellow shift and dramatic reduction in color rendering properties of the mouse-matched spectrum. (**D**–**F**) Spectral power densities (left), α-opic EDIs (top right) for human (gray), and mouse (magenta); and associated color properties (as **C**; bottom right) of 3 additional white light sources, which provide progressively better approximations of natural daylight for mice. Panels, respectively, represent a standard 4000K fluorescent source (**D**), a high-quality 6500K “daylight” fluorescent (**E**), and a violet-pumped LED source (**F**), modeled based on commercially available devices. EDI, equivalent daylight illuminance.

Unlike wavelength, which is a physical property, color is a perceptual quality whose biological origins reflect the differential stimulation across classes of retinal opsins, (principally those expressed by cone photoreceptors [76]), and subsequent signal processing. As a result, differences in cone spectral sensitivity can substantially skew an animal’s experience of color relative to our own. Most mammals possess just 2 of the 3 cone opsin types found in humans (an S-cone opsin and an M/L-cone opsin), limiting them to a single dimension of color discrimination [77,78]. Moreover, in mice and several other rodent species, the spectral sensitivity of these 2 cone types (and corresponding capacity for color discrimination [79–84]) is substantially short-wavelength shifted relative to their human counterparts, with the S-opsin showing maximal sensitivity at wavelengths that are largely undetectable to humans (peak sensitivity approximately 360 nm) [85]. As a result, most common light sources, which lack energy in this part of the spectrum (particularly commonly used white LEDs), are expected to appear as extremely long-wavelength biased compared to daylight (“yellow” by human analogy) for most mammals and to dramatically limit any capacity for color discrimination. The α-opic metrology allows quantification of this property (Fig 4).

The importance of light’s spectral properties for mammalian health, physiology, and behavior is incompletely understood. Across non-mammalian species, there are many demonstrations that appropriate spectral content is critical for key behaviors including navigation, hunting, and mating [86–91]. Similarly, there is evidence that the short-wavelength-shifted spectral discrimination capacity of mice and other rodents is important for foraging, social/territorial, and/or defensive behaviors [92–95]. There is also growing evidence that color signals contribute to the circadian control of physiology and behavior by providing information about shifts in the spectral composition of ambient light occurring during twilight [96]. Indeed, it is now apparent that, in mice, spectral signals originating from cones influence neural activity within the master circadian clock (the suprachiasmatic nucleus) and can modulate the timing and robustness of behavioral and physiological rhythms [5,97]. Finally, there are emerging data of a protective role of “violet” (360 to 400 nm) light against the development of myopia in mice and other species via, as yet incompletely resolved, mechanisms [29,30,41,98–101].

In sum, while there is much we do not know about the biological importance of the spectrum of light, there is reason to suspect that failure to provide an approximation of the animal’s experience of natural light could alter species-specific behaviors, circadian function, and aspects of development. It is generally accepted that any restrictions on the extent to which animals can satisfy their physiological or ethological needs should be kept to a minimum [102], so there is a clear ethical requirement to approximate natural light, besides the likely benefits for animal welfare (see below) and data quality. In addition to encouraging more research in this area, we note that there may be opportunities to better recreate the animal’s experience of the spectral properties of natural daylight (including considerations around the spectral transmission of cages/enclosures where relevant). In the case of mice and many other commonly used laboratory rodents, this could be simply achieved without compromising the experience of humans in the same environment by choosing fluorescent or LED lighting with greater energy in short wavelength portions (390 to 420 nm) of the visible spectrum (Fig 4E and 4F). A violet pumped LED would provide such a lighting solution, providing a “white-light” perception for both humans and rodents that approaches the perception of natural sunlight for most mammals (Fig 4F).

### Animal welfare implications

The quantitative guidance we presented on animal husbandry represents the first specification targeting the animal experience of light. To our knowledge, the animal welfare implications of inappropriate light quality in vivarium housing are yet to be specifically evaluated. There is, however, a body of experimental literature on the effects of ocular light exposure on specific elements of health and wellbeing, which can be used to identify potential risks to animal welfare.

Disruption of circadian rhythms is known to be detrimental to health in humans and may contribute to a range of different diseases [103,104]. Given that rodents are used to model the effects of circadian disruption in humans, to help understand deleterious effects on humans and how these might be ameliorated, it is not surprising that light also affects animal welfare [105–107]. There is a large body of evidence on the effects of circadian disruption on animal health. In rodents, exposure to non-24 h light/dark cycles reduced lifespan, and this effect was abolished in constant darkness [108,109]. The health consequences of circadian disruption have been studied in rodents under many different experimental conditions [110,111]. For example, exposure to light at night impairs activity/rest cycles and blunts glucocorticoid rhythms [112], affects metabolism [113] and immune function [114], and increases anxiety and depression-like behaviors in a wavelength-dependent manner [115,116]. Aberrant light/dark cycles also impair learning, memory, and mood in mice [21]. Even conditions that produce a misalignment of circadian phase by a few hours can alter cardiometabolic function, sleep, and recognition memory [117,118].

Together, these data provide strong evidence that disrupting circadian rhythms via inappropriate light exposure can have detrimental effects on animal health and welfare. Exposure to short-wavelength-enriched light during the daytime may provide benefits for circadian, metabolic, and endocrine regulation [69], though more data are needed regarding the optimum intensity and spectral composition of normal vivarium lighting.

## Messaging to stakeholders and effecting change

The concept of redefining how light is measured and reported (and, ultimately, provided) will be new to many stakeholders. Reporting light parameters in detail, beyond basic information on light/dark phases, will also likely be a novel approach. Given that there have been issues with the impact of the widely supported, and promoted, ARRIVE 2.0 guidelines on reporting animal use [119], it is likely that considerable effort will have to be put into communicating with stakeholders and persuading them to report lighting appropriately. One important determinant of success will be cost/benefit calculations. On the cost side, we hope that methods presented here will make species-specific light measurement accessible for many and that appropriate light meters will become increasingly available. That will be important if the new measurement system is to be accessible to those without specialist knowledge (or interest) in light. Turning to benefits, it is important to highlight what could be gained by quantifying and specifying light according to the animal’s experience, contributing to animal welfare and hence, the quality of scientific output.

### Scientific researchers

**Fig 5:**
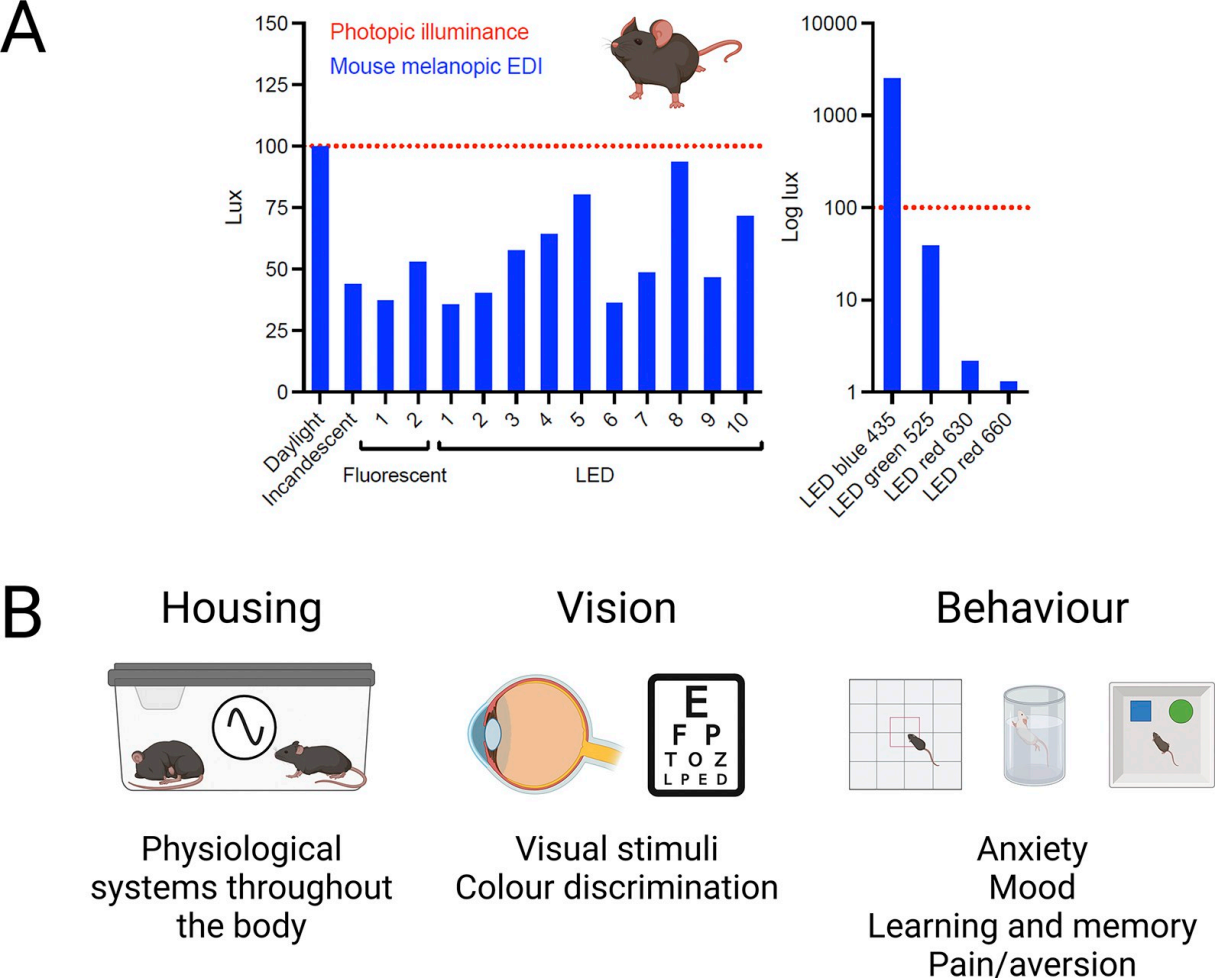
The importance of measuring light in appropriate species-specific metrics. (**A**) Illustration of the inaccuracy of measuring light in photopic lux. Broad-band (white) light sources of equal photopic lux give very different mouse melanopic EDI values depending upon their spectral content. This difference becomes even larger for narrow-band (colored) light sources (note log scale). For example, blue LEDs can give higher melanopic EDI values, whereas red LEDs give much lower values. (**B**) Appropriate α-opic measurements should be considered for routine housing and husbandry, as inappropriate light exposure may disrupt daily variations in behavioral and physiological state, with consequences for many aspects of biology. These metrics will also facilitate recreation of experimental conditions for any study in which light is an independent variable, including vision and circadian research. Finally, α-opic EDI measurements represent a first step in standardizing experimental conditions for any work in which light can impact the outcome, including common behavioral tests of anxiety, mood, learning and memory, and pain/aversion. Created with BioRender.com. EDI, equivalent daylight illuminance.

When light is itself an experimental parameter, the benefits of proper quantification should be self-evident. “White” lights matched for photopic lux can vary by as much as 3-fold in mouse melanopic irradiance. The difference becomes larger for “colored” lighting; for example, mouse melanopic irradiance of a “blue” 435 nm light could be 60 or 2,500 times greater than that of a “green” or “red” light with the same photopic lux (Fig 5A). Thus, the animal experience of different lights matched in inappropriate quantities could be very divergent, leading to inappropriate scientific conclusions and failures of replication.

There are many experiments in which light is an aspect of the wider experimental conditions that could influence the outcome. Given the evidence that light can have wide-ranging effects on behavioral and physiological states, standardizing light intensity in units appropriate for the species could improve reproducibility in several ways (Fig 5B).

The most wide-ranging way in which appropriate light measurement could improve scientific outcomes is in standardizing the measurement of housing conditions and avoiding bad practice. As described above, light has profound effects on physiology and behavior. Exposure to inappropriate light in the “night” phase can impact numerous aspects of biology, from activity/rest cycles to hormone levels and cognitive function [113,120]. Light levels also vary dramatically across cage racks [52], and animals housed under different lighting conditions will start from a different baseline. By adhering to the quantitative guidance provided here for animal husbandry prior to and during experimentation, researchers can avoid bad practices (e.g., excessive light at night) that have the potential to impact their scientific data. The α-opic EDI metrology we propose also enables researchers to avoid systematic biases in their experimental design. For example, defining variations in lighting that occur across cage racks or within rooms allow treatment groups to be appropriately counterbalanced [121]. Analysis of data in this manner also offers a powerful approach to identify how light—measured in species-appropriate units—affects commonly measured experimental outcomes.

Light is also critical for visual function, and appropriate light measurement is essential for any studies where visual stimuli are used, whether this involves pattern recognition, visual acuity, movement detection, changes in brightness, or visually guided behaviors. Differences in the tuning of color discrimination across species could also be relevant for many assays of animal behavior and cognition that involve a visual component (for example, novel object recognition). In many such cases, the choice of cue and lighting properties could substantially impact the extent to which the relevant visual cues are distinguishable to the animal. This may alter the sensitivity and reliability of the test in question (for example, by varying across a “green–red” axis rather than the “violet–green” axis, across which mice can discriminate color). The use of α-opic metrics provides a simple way of capturing and standardizing these aspects of the animal experience.

Properly quantifying light would improve reproducibility in behavioral studies, including tests of anxiety, mood, and learning and memory [122]. Many tests of anxiety in rodents depend upon photophobia, such as the open field test, elevated plus maze, and light/dark box [71]. Moreover, as light has been shown to modulate mood, learning, and memory [19,21], differences in lighting between studies may provide an experimental confound. Recent data showing that pain is affected by light [24,25] are relevant not only to pain research, but also to any condition where animals undergo invasive procedures where light may influence an animal’s subsequent recovery. The use of α-opic metrics should help reproducibility of scientific data both within and between labs.

### Building designers and facility managers

Quantitative specifications provide the substrate for engineering. We hope that defining what animals need will precipitate innovative solutions to achieve it. These could lead to cost savings and improvements in energy efficiency, but one particularly attractive target will be resolving potential conflicts between the needs of humans and lab animals occupying the same environment. One example of such an application would be adoption of lighting that approximates the experience of daylight for both species (Fig 4). Another example with strong potential is to facilitate reverse light/dark cycles. This can be an important strategy for allowing data collection during a nocturnal rodent’s active phase [123], but imposes a conflict between the lighting needs of animals and their human caretakers. Genuinely reversed light/dark cycles, in facilities where this has not been specifically designed and facilitated, can be difficult to achieve and are easily undermined by even very low, or fleeting, exposures to light. The animal-centric guidance provided here can be applied to employing “deep red” light to resolve this conflict. Thus, for example, applying melanopic irradiance reveals that >680 nm task/room lighting (appearing red to humans), may be as bright as 300 photopic lux without exceeding the limit for nocturnal light exposure (<0.1 lx melanopic EDI) in lab mammals proposed here. Continuous dim red safety lighting is not recommended, however, as such chronic nocturnal light exposure may affect circadian physiology [124].

How should those involved in the design and management of facilities proceed? Due to the highly regulated environment necessary in animal facilities, energy usage—and the associated costs—are a major concern. Many facilities are justifiably moving to more energy efficient LEDs to replace older lighting systems. Where LED lighting is used, we would recommend adoption of lighting systems that can be made to approximate daylight for both humans and lab animals. For rodents, this may involve the future capacity to use violet-pumped white LEDs, which would be necessary to approximate daylight in species with S-cones sensitive to ultraviolet light. While there are little empirical data on the behavioral consequences of such daylight approximation, selection of lighting systems that are flexible and accommodate tunable spectral output may be desirable.

## Conclusions

The current practice of measuring light using units designed against a human observer leaves this important environmental parameter poorly controlled in animal husbandry and experimentation. Light sources that appear similar to humans may appear quite different to animals. It is important to be aware of the potential consequences for animal welfare, reduction and refinement, and data quality, if lighting is inappropriate. Measuring light in species-specific α-opic EDI provides a workable approach to quantifying the full animal experience of light. The newly standardized melanopic EDI unit represents the best currently available single measure for ambient light intensity across mammalian species. We provide targets for light exposure in melanopic EDI in husbandry in the animal’s daytime and night. Until it is technically and practically feasible for these targets to be achieved, it is essential that light levels and quality are effectively reported in publications.

## Supporting information

S1 Text Further considerations and assumptions in defining *s*_*αβ*_(*λ*). Extended description of the approach used to define spectral efficiency functions *s*_*αβ*_(*λ*), and the particular challenge of doing so for OPN3 and OPN5.
(DOCX)

S1 Table α-opic sensitivity functions for retinal photopigments in some common laboratory species. (XLSX)

S2 Table A selection of light meters and spectrometers suitable for α-opic EDI measurement. (XLSX)

S3 Table Comparison of mouse α-opic EDIs across 42 broad-spectrum CIE standard white light sources matched for 100 human photopic lx. (XLSX)

S4 Table Comparison of mouse α-opic EDIs across 9 monochromatic LED light sources matched for 100 human photopic lx. (XLSX)

## Abbreviations

CIE: International Committee on Illumination
EDI: equivalent daylightilluminance
ELR: efficacy of luminous radiation
ipRGC: intrinsically photosensitive retinal ganglion cell
lx: lux
mel: melanopic
MWS: medium-wavelength sensitive
Rho: rhodopic
SI: System International
SWS: short-wavelength sensitive
