# Supplementary material for: Recommendations for measuring and standardizing light for laboratory mammals to improve welfare and reproducibility in animal research
Source: PLoS Biol. 2024 Mar 12;22(3):e3002535. doi: 10.1371/journal.pbio.3002535 (PMC10931507; doi:10.1371/journal.pbio.3002535)
Supplement: S1 Text — Extended description of the approach used to define spectral efficiency functions sαβ(λ), and the particular challenge of doing so for OPN3 and OPN5. (DOCX) [file pbio.3002535.s001.docx]

**Supplementary text – Notes on defining** $\boldsymbol{s}_{\boldsymbol{\alpha\beta}}\left( \boldsymbol{\lambda} \right)$**:**

The α-opic metrology relies critically on the quality of the underlying spectral normalisation functions $s_{\alpha\beta}\left( \lambda\right)$. Here, we describe some of the considerations and assumptions in deriving these functions.

A first consideration is within-species, inter-individual, variation. While acknowledging that this could be substantial, we suggest adopting the concept of a standard observer, originally developed in the field of human colour vision research. In that context, a standard observer is a hypothetical, typical human with a healthy visual system that is described by mathematical equations relating to visual perception of quantifiable light stimuli. In the context of this paper, the standard observer would be a healthy adult representative of a specific mammalian species with an intact, healthy eye. For example, a standard mouse observer might be a healthy mature adult, aged 3-6 months, of the widely used pigmented inbred strain C57BL/6J.

In developing $s_{\alpha\beta}\left( \lambda\right)$ it is important to remember that *in vivo* spectral sensitivity is determined by two factors - the photopigment’s intrinsic wavelength preference ($a_{\alpha\beta}\left( \lambda\right));$and any spectral filtering of light as it passes through the eye ($p_{\alpha\beta}\left( \lambda\right)$) – according to equation A:

$E_{\alpha\beta}=\int E_{e,\lambda}\left( \lambda\right)a_{\alpha\beta}\left( \lambda\right) p_{\alpha\beta}\left( \lambda\right)d\lambda$ Equation A

Where $E_{\alpha\beta}$ is the α-opic irradiance, defined as the effective irradiance for given photopigment (α) in a given species (β), with units in W/m^2^; and $E_{e,\lambda}$the measured spectral irradiance, with units W/m^2^. Where photopigment absorption spectra are expressed on a per photon rather than a per energy basis, to give radiometric α-opic sensitivity functions, *in vivo* spectral sensitivity is also corrected by the energy per photon (hc/λ) and is expressed relative to the peak sensitivity (**S1 Table**).

Photopigment spectral sensitivity ($a_{\alpha\beta}\left( \lambda\right))$

A pigment’s photosensitivity is a product of the probability of it absorbing a photon (its extinction coefficient, which varies with wavelength), and the probability that photon absorption activates it (its quantum efficiency of isomerization, which does not vary with wavelength) [1]. For all pigments that use a chromophore derived from vitamin A (which includes rhodopsin, cone opsins, and melanopsin), the resultant spectral sensitivity ($a_{\alpha\beta}\left( \lambda\right)$) is well-predicted by an empirically derived equation, generally referred to as the visual pigment template or nomogram [2, 3]. The equations of Govardovskii and colleagues are the standard; with only the wavelength of maximum sensitivity λ_max_ as a free parameter, they produce the full spectral sensitivity of a photopigment with high accuracy [4]. Variations in quantum efficiency do not change λ_max_ but may alter effective sensitivity between pigments. The quantum efficiency of isomerization is unknown except for a few opsins, although it is probably safe to assume that it is within the measured range of 0.4 - 0.7 for mammalian opsins [3, 5-7]. For simplicity, our definitions of $a_{\alpha\beta}\left( \lambda\right)$assume equivalent quantum efficiency across opsins.

The λ_max_ values of cone pigments have been determined for many species and span a broad range [8]. The mouse, for example, has two cone pigments with λ_max_ values near 508 and 358 nm [6, 7]. There appears to be remarkably little variation in spectral sensitivity for rhodopsin between mammalian species [9, 10], with the exception of deep diving mammals [11]. As a result, in species for which there are no existing data, the λ_max_ of rhodopsin can generally be inferred to be ~500 nm.

Melanopsin’s spectral sensitivity also appears stable across mammalian species, being describable by a pigment template with a λ_max_ of 480 nm [12-17]. This λ_max_ adequately describes the spectral sensitivity of melanopsin-driven responses in mice, rats and macaques, and has also proven effective for predicting melanopsin-driven responses in humans [12, 17-20].

That said, this λ_max_ value has a complex origin. While rhodopsin and cone opsins each activate from one state, mammalian melanopsin can activate from two states: melanopsin (R) and extramelanopsin (E) [5, 21-23]. These states have different spectral sensitivities (λ_max_ values of 490 and 470 nm, respectively) and the spectral sensitivity of cellular activation reflects their combination, which is a broadened spectrum with a peak at 480 nm. Most white light sources, both natural and artificial, will cause melanopsin molecules to activate about evenly from the R and E states [22]. This broadening causes a ~3% increase in melanopsin activation under sunlight and will have a stronger impact under short-wavelength illumination. Another complexity is that, while rhodopsin and cone opsins disassemble shortly after activation, melanopsin’s signalling state (metamelanopsin, M, λ_max_ = 476 nm) is highly stable. When produced in sufficient quantity, it produces robust cellular activation that persists for minutes even after illumination ceases. This persistent activity can be acutely curtailed by illumination with wavelengths of ~550 nm or longer, which drive melanopsin to the non-signaling E state. Extraordinarily high photon fluxes (e.g., 10^9^ photons µm^-2^ s^-1^) are required because all melanopsin states absorb these wavelengths poorly [22-24]. Such photon fluxes are unlikely to be encountered in the environment but can be produced artificially to suppress melanopsin activity. To summarize, melanopsin’s multistable nature gives rise to spectral broadening and photoswitchable persistent activity, features that are not found in rhodopsin and cone opsins.

Incidentally, the λ_max_ ­­values for melanopsin that are noted here differ from some reported previously [5, 22, 23]. These prior studies tested melanopsin’s spectral sensitivity under conditions designed to isolate the R or E state. They also fit pigment templates to those data on a linear ordinate, which tends to give a shorter λ_max_. By contrast, most assessments of melanopsin spectral sensitivity have not sought to separate R and E states and thus likely reflect their combined activity, and fit nomograms on a log ordinate to give a longer λ_max_ [12, 17-19]. Given the prevalence of the latter measurements, it is generally recommended to use their value for melanopsin activation (λ_max_ = 480 nm).

Note that there are many ways to measure spectral sensitivity. It is possible to measure spectral sensitivity *in vivo* using techniques such as the electroretinogram (ERG). *In vivo* measurement of spectral sensitivity generally negates the need for further characterization of pre-receptoral filtering, in the context of this review, as this is implicitly accounted for [25, 26]. Alternatively, *ex vivo* retinal tissue can be used to perform electrophysiology or microspectrophotometry [27] on individual cells. With the advent of readily available sequenced genomes, it is possible to recombinantly express opsin genes in cell lines and use these to generate action spectra in live cells [17], or purify pigment for spectrophotometry. Each of these techniques introduces a degree of variance into the measurement of spectral sensitivity. In general, the variation is probably negligible outside of a vision research laboratory, though exceptions exist (the spectral sensitivity of a pigment depends on its chromophore, of which there are several kinds and isomers, and on additional parameters such as the pH and ionic composition of the medium) [4, 28, 29].

Pre-receptoral filtering ($a_{\alpha\beta}\left( \lambda\right)$)

Turning to the second determinant of $S_{\alpha\beta}$, the mammalian eye’s light path has several components that could alter spectral transmission: the cornea, aqueous humor, iris, lens, vitreous humor, and retina. In addition, some species have a tapetum lucidum behind the retina, which reflects light back through the retina. Each ocular element may alter light, such as by reflection, scatter, and absorption. These alterations may vary with species, retinal location, and age. The human eye provides an example. The highly specialized centre of its retina (the fovea) attenuates short wavelengths by expressing an absorbing pigment, clarifies the visual image by excluding blood vessels, and provides photoreceptors with direct access to that image by displacing overlaying retinal layers [30]. The peripheral retina lacks these features. Thus, given an identical image, photopigments in the central and peripheral retina activate differently. Moreover, these features of the central retina are unique to several primate species, being absent in other mammals. To learn how light affects an animal, one must know how light is shaped in the path from its source to the relevant photopigments.

With this reminder that there are circumstances in which a more complete understanding of pre-receptoral filtering is critical, in the absence of full information about all components we argue that it is acceptable to take lens transmission as the primary determinant of $p_{\alpha\beta}\left( \lambda\right)$for common lab species. The aqueous and vitreous humor appear largely transparent and are often ignored. Hence, the cornea and lens are the main filters in front of the retina. The iris is generally considered opaque; some light does penetrate this tissue but is likely negligible compared to that passing through the open pupil. Cornea and lens are long-pass filters: transmission increases from ultraviolet to infrared. At 500 nm, where the rod photoreceptor (and thus the animal) is most sensitive, each cuts ~30% of the incoming photons in mouse [31]. Lens absorption spectra show substantial variation between species [8, 32].

In common with most studies, we assume that spectral filtering by the retina is negligible. This is likely a reasonable assumption for the intrinsically photosensitive retinal ganglion cells (ipRGCs) whose somata lie near the surface and dendrites stratify <100 µm below [33-35]. Retinal filtering may be more substantial for the rods and cones, which lie farthest from the incoming light. The parameters of filtering are unknown for the mouse but are likely similar to those of the rat, which cut shorter wavelengths more than longer ones, attenuating ~30% at 500 nm [36]. Other features of the retina that can be important but are generally ignored include additional filtering by blood vessels [25] and specializations for transmitting light, such as the transparency of cell nuclei, lensing by mitochondria, and waveguiding [37-40]. A final note of caution is appropriate with regard to marsupials, which can have oil droplets as additional spectral filters in their retinae [41, 42].

Light diminishes further as it propagates through the photoreceptor cell and is absorbed by photopigments. This “self-screening” causes photopigments at the far (outer) end of the mouse rod photoreceptor to receive ~35% less light than those at the near (inner) end [43]. As such self-screening is, by definition, not spectrally neutral; it can influence effective spectral sensitivity at high photon flux. However, as the degree of such self-screening is not generally known for most animal photoreceptors we have not considered it in our descriptions of photoreceptor spectral sensitivity.

Light that escapes capture by the photoreceptor cells reaches the retinal pigment epithelium (RPE), which in pigmented animals expresses melanin and absorbs visible light effectively. In many mammalian species the reflective tapetum lucidum may apply an additional modulation of spectral quality of light reaching the photoreceptor, but this is not considered in our current definition of $s_{\alpha\beta}\left( \lambda\right).$

**Opn3 and Opn5**

The potential of Opn3 and Opn5 to function outside the retina complicates defining α-opic quantities. As noted above, $s_{\alpha\beta}\left( \lambda\right)$ is a product of both photopigment spectral efficiency function and pre-receptoral filtering. Outside the retina, pre-receptoral filtering would be a substantial influence and would vary according to where the pigment is expressed. For this reason, it is substantially more difficult to extend the α-opic concept to extra-retinal photoreception. Accordingly, we do not specify metrics for Opn3 or Opn5 here. We do support future work applying the a-opic concepts to resolve this omission as appropriate (based upon both pigment absorption and relevant pre-receptor filtering) and look forward to seeing their wider adoption as the field matures. As an aside, for functions within the eye, we note that the available information indicates peak sensitivity around 380nm for Opn5 [44] (the spectral sensitivity of Opn3 is not currently defined although evidence suggests short wavelength sensitivity), meaning that under most circumstances it will strongly co-vary with S-cone opic irradiance.

**REFERENCES**

1. Fein A, Szuts EZ. Photoreceptors: Their Role in Vision: Cambridge University Press; 1982.

2. Lamb TD. Photoreceptor spectral sensitivities: common shape in the long-wavelength region. Vision research. 1995;35(22):3083-91. Epub 1995/11/01. PubMed PMID: 8533344.

3. Dartnall HJA. Photosensitivity. In: Dartnall HJA, editor. Photochemistry of Vision. Handbook of Sensory Physiology. VII/1. New York: Springer-Verlag; 1972. p. 122-45.

4. Govardovskii VI, Fyhrquist N, Reuter T, Kuzmin DG, Donner K. In search of the visual pigment template. Vis Neurosci. 2000;17(4):509-28. Epub 2000/10/04. PubMed PMID: 11016572.

5. Matsuyama T, Yamashita T, Imamoto Y, Shichida Y. Photochemical Properties of Mammalian Melanopsin. Biochemistry. 2012;51:5454-62. Epub 2012/06/08. doi: 10.1021/bi3004999. PubMed PMID: 22670683.

6. Sakurai K, Onishi A, Imai H, Chisaka O, Ueda Y, Usukura J, et al. Physiological properties of rod photoreceptor cells in green-sensitive cone pigment knock-in mice. J Gen Physiol. 2007;130(1):21-40. Epub 2007/06/27. doi: 10.1085/jgp.200609729. PubMed PMID: 17591985; PubMed Central PMCID: PMCPMC2154367.

7. Tsutsui K, Imai H, Shichida Y. Photoisomerization efficiency in UV-absorbing visual pigments: protein-directed isomerization of an unprotonated retinal Schiff base. Biochemistry. 2007;46(21):6437-45. Epub 20070503. doi: 10.1021/bi7003763. PubMed PMID: 17474760.

8. McDowell RJ, Didikoglu A, Woelders T, Gatt MJ, Brown TM, Lucas RL. Beyond Lux: Methods for Species and Photoreceptor-Specific Quantification of Ambient Light for Domestic Mammals. Biorxiv. 2023. doi: <https://doi.org/10.1101/2023.08.25.554794>.

9. Zhao H, Ru B, Teeling EC, Faulkes CG, Zhang S, Rossiter SJ. Rhodopsin molecular evolution in mammals inhabiting low light environments. PLoS One. 2009;4(12):e8326. Epub 2009/12/18. doi: 10.1371/journal.pone.0008326. PubMed PMID: 20016835; PubMed Central PMCID: PMCPMC2790605.

10. Bridges CD. Visual pigments of some common laboratory mammals. Nature. 1959;184(Suppl 22):1727-8. doi: 10.1038/1841727a0. PubMed PMID: 13804386.

11. Fasick JI, Robinson PR. Spectral-tuning mechanisms of marine mammal rhodopsins and correlations with foraging depth. Vis Neurosci. 2000;17(5):781-8. Epub 2001/01/12. doi: 10.1017/s095252380017511x. PubMed PMID: 11153657.

12. Lucas RJ, Douglas RH, Foster RG. Characterization of an ocular photopigment capable of driving pupillary constriction in mice. Nat Neurosci. 2001;4(6):621-6. doi: 10.1038/88443. PubMed PMID: 11369943.

13. Tu DC, Zhang D, Demas J, Slutsky EB, Provencio I, Holy TE, et al. Physiologic diversity and development of intrinsically photosensitive retinal ganglion cells. Neuron. 2005;48(6):987-99. Epub 2005/12/21. doi: 10.1016/j.neuron.2005.09.031. PubMed PMID: 16364902.

14. Panda S, Nayak SK, Campo B, Walker JR, Hogenesch JB, Jegla T. Illumination of the melanopsin signaling pathway. Science. 2005;307(5709):600-4. Epub 2005/02/01. doi: 10.1126/science.1105121. PubMed PMID: 15681390.

15. Gamlin PD, McDougal DH, Pokorny J, Smith VC, Yau KW, Dacey DM. Human and macaque pupil responses driven by melanopsin-containing retinal ganglion cells. Vision research. 2007;47(7):946-54. Epub 2007/02/27. doi: 10.1016/j.visres.2006.12.015. PubMed PMID: 17320141; PubMed Central PMCID: PMCPMC1945238.

16. Hattar S, Lucas RJ, Mrosovsky N, Thompson S, Douglas RH, Hankins MW, et al. Melanopsin and rod-cone photoreceptive systems account for all major accessory visual functions in mice. Nature. 2003;424(6944):76-81. Epub 2003/06/17. doi: 10.1038/nature01761. PubMed PMID: 12808468; PubMed Central PMCID: PMCPMC2885907.

17. Bailes HJ, Lucas RJ. Human melanopsin forms a pigment maximally sensitive to blue light (lambdamax approximately 479 nm) supporting activation of G(q/11) and G(i/o) signalling cascades. Proc Biol Sci. 2013;280(1759):20122987. Epub 2013/04/05. doi: 10.1098/rspb.2012.2987. PubMed PMID: 23554393; PubMed Central PMCID: PMCPMC3619500.

18. Dacey DM, Liao HW, Peterson BB, Robinson FR, Smith VC, Pokorny J, et al. Melanopsin-expressing ganglion cells in primate retina signal colour and irradiance and project to the LGN. Nature. 2005;433(7027):749-54. Epub 2005/02/18. doi: 10.1038/nature03387. PubMed PMID: 15716953.

19. Berson DM, Dunn FA, Takao M. Phototransduction by retinal ganglion cells that set the circadian clock. Science. 2002;295(5557):1070-3. Epub 2002/02/09. doi: 10.1126/science.1067262. PubMed PMID: 11834835.

20. Brown TM. Melanopic illuminance defines the magnitude of human circadian light responses under a wide range of conditions. J Pineal Res. 2020;69(1):e12655. Epub 20200419. doi: 10.1111/jpi.12655. PubMed PMID: 32248548.

21. Emanuel AJ, Do MT. The multistable melanopsins of mammals. Front Ophthalmol. 2023;3. Epub 26 April 2023. doi: <https://doi.org/10.3389/fopht.2023.1174255>.

22. Emanuel AJ, Do MTH. Melanopsin tristability for sustained and broadband phototransduction. Neuron. 2015;85(5):1043-55. Epub 2015/03/06. doi: 10.1016/j.neuron.2015.02.011. PubMed PMID: 25741728; PubMed Central PMCID: PMC4351474.

23. Liu A, Milner ES, Peng YR, Blume HA, Brown MC, Bryman GS, et al. Encoding of environmental illumination by primate melanopsin neurons. Science. 2023;379(6630):376-81. Epub 2023/01/27. doi: 10.1126/science.ade2024. PubMed PMID: 36701440.

24. Emanuel AJ, Kapur K, Do MTH. Biophysical Variation within the M1 Type of Ganglion Cell Photoreceptor. Cell Reports. 2017;21:14.

25. Spitschan M, Aguirre GK, Brainard DH. Selective stimulation of penumbral cones reveals perception in the shadow of retinal blood vessels. PLoS One. 2015;10(4):e0124328. Epub 2015/04/22. doi: 10.1371/journal.pone.0124328. PubMed PMID: 25897842; PubMed Central PMCID: PMCPMC4405364.

26. Snodderly DM, Brown PK, Delori FC, Auran JD. The macular pigment. I. Absorbance spectra, localization, and discrimination from other yellow pigments in primate retinas. Investigative ophthalmology & visual science. 1984;25(6):660-73. Epub 1984/06/01. PubMed PMID: 6724836.

27. Bowmaker JK. Microspectrophotometry of vertebrate photoreceptors. A brief review. Vision research. 1984;24(11):1641-50. Epub 1984/01/01. doi: 10.1016/0042-6989(84)90322-5. PubMed PMID: 6398563.

28. Yamashita T, Nakamura S, Tsutsui K, Morizumi T, Shichida Y. Chloride-dependent spectral tuning mechanism of L-group cone visual pigments. Biochemistry. 2013;52(7):1192-7. Epub 2013/01/29. doi: 10.1021/bi3016058. PubMed PMID: 23350963.

29. Isayama T, Alexeev D, Makino CL, Washington I, Nakanishi K, Turro NJ. An accessory chromophore in red vision. Nature. 2006;443(7112):649. Epub 2006/10/13. doi: 10.1038/443649a. PubMed PMID: 17035994.

30. Provis JM, Dubis AM, Maddess T, Carroll J. Adaptation of the central retina for high acuity vision: cones, the fovea and the avascular zone. Progress in retinal and eye research. 2013;35:63-81. Epub 2013/03/19. doi: 10.1016/j.preteyeres.2013.01.005. PubMed PMID: 23500068; PubMed Central PMCID: PMC3658155.

31. Henriksson JT, Bergmanson JP, Walsh JE. Ultraviolet radiation transmittance of the mouse eye and its individual media components. Exp Eye Res. 2010;90(3):382-7. Epub 2009/11/21. doi: 10.1016/j.exer.2009.11.004. PubMed PMID: 19925789.

32. Douglas RH, Jeffery G. The spectral transmission of ocular media suggests ultraviolet sensitivity is widespread among mammals. Proc Biol Sci. 2014;281(1780):20132995. Epub 20140219. doi: 10.1098/rspb.2013.2995. PubMed PMID: 24552839; PubMed Central PMCID: PMCPMC4027392.

33. Berson DM, Castrucci AM, Provencio I. Morphology and mosaics of melanopsin-expressing retinal ganglion cell types in mice. Journal of Comparative Neurology. 2010;518(13):17. doi: 10.1002/cne.22381.

34. Quattrochi LE, Stabio ME, Kim I, Ilardi MC, Michelle Fogerson P, Leyrer ML, et al. The M6 cell: A small-field bistratified photosensitive retinal ganglion cell. The Journal of comparative neurology. 2018. Epub 2018/10/13. doi: 10.1002/cne.24556. PubMed PMID: 30311650.

35. Do MTH. Melanopsin and the Intrinsically Photosensitive Retinal Ganglion Cells: Biophysics to Behavior. Neuron. 2019;104(2):205-26. doi: 10.1016/j.neuron.2019.07.016. PubMed PMID: 31647894; PubMed Central PMCID: PMCPMC6944442.

36. Alpern M, Fulton AB, Baker BN. "Self-screening" of rhodopsin in rod outer segments. Vision research. 1987;27(9):1459-70. Epub 1987/01/01. doi: 10.1016/0042-6989(87)90155-6. PubMed PMID: 3445480.

37. Solovei I, Kreysing M, Lanctot C, Kosem S, Peichl L, Cremer T, et al. Nuclear architecture of rod photoreceptor cells adapts to vision in mammalian evolution. Cell. 2009;137(2):356-68. Epub 2009/04/22. doi: 10.1016/j.cell.2009.01.052. PubMed PMID: 19379699.

38. Franze K, Grosche J, Skatchkov SN, Schinkinger S, Foja C, Schild D, et al. Muller cells are living optical fibers in the vertebrate retina. Proceedings of the National Academy of Sciences of the United States of America. 2007;104(20):8287-92. Epub 2007/05/09. doi: 10.1073/pnas.0611180104. PubMed PMID: 17485670; PubMed Central PMCID: PMC1895942.

39. Ball JM, Chen S, Li W. Mitochondria in cone photoreceptors act as microlenses to enhance photon delivery and confer directional sensitivity to light. Sci Adv. 2022;8(9):eabn2070. Epub 2022/03/03. doi: 10.1126/sciadv.abn2070. PubMed PMID: 35235359; PubMed Central PMCID: PMCPMC8890704.

40. Naarendorp F, Esdaille TM, Banden SM, Andrews-Labenski J, Gross OP, Pugh EN, Jr. Dark light, rod saturation, and the absolute and incremental sensitivity of mouse cone vision. The Journal of neuroscience : the official journal of the Society for Neuroscience. 2010;30(37):12495-507. Epub 2010/09/17. doi: 10.1523/JNEUROSCI.2186-10.2010. PubMed PMID: 20844144; PubMed Central PMCID: PMC3423338.

41. Arrese CA, Hart NS, Thomas N, Beazley LD, Shand J. Trichromacy in Australian marsupials. Curr Biol. 2002;12(8):657-60. Epub 2002/04/23. doi: 10.1016/s0960-9822(02)00772-8. PubMed PMID: 11967153.

42. O'Day K. A Preliminary Note on the Presence of Double Cones and Oil Droplets in the Retina of Marsupials. J Anat. 1936;70(Pt 4):465-7. Epub 1936/07/01. PubMed PMID: 17104608; PubMed Central PMCID: PMCPMC1249140.

43. Lyubarsky AL, Daniele LL, Pugh EN, Jr. From candelas to photoisomerizations in the mouse eye by rhodopsin bleaching in situ and the light-rearing dependence of the major components of the mouse ERG. Vision research. 2004;44(28):3235-51. doi: 10.1016/j.visres.2004.09.019. PubMed PMID: 15535992.

44. Kojima D, Mori S, Torii M, Wada A, Morishita R, Fukada Y. UV-sensitive photoreceptor protein OPN5 in humans and mice. PLoS One. 2011;6(10):e26388. Epub 2011/11/02. doi: 10.1371/journal.pone.0026388. PubMed PMID: 22043319; PubMed Central PMCID: PMCPMC3197025.
